# Immersive virtual reality in children with upper limb injuries: Findings from a feasibility study

**DOI:** 10.3233/PRM-190635

**Published:** 2021-09-28

**Authors:** Ivan Phelan, Penny J. Furness, Heather D. Dunn, Alicia Carrion-Plaza, Maria Matsangidou, Paul Dimitri, Shirley Lindley

**Affiliations:** a Centre for Culture, Media and Society, College of Social Sciences and Arts, Sheffield Hallam University, Sheffield, United Kingdom; b Centre for Behavioural Sciences and Applied Psychology (CeBSAP), Department of Psychology, Sociology and Politics, College of Social Sciences and Arts, Sheffield Hallam University, Sheffield, United Kingdom; c NIHR Children & Young People MedTech Cooperative, Sheffield Children’s NHS Foundation Trust, Sheffield, United Kingdom; d Sheffield Hallam University, Sheffield, United Kingdom

**Keywords:** Virtual reality, upper limb injury, children’s rehabilitation, physiotherapy, burns, fractures

## Abstract

**PURPOSE:**

Children who sustain Upper Limb Injuries (ULIs), including fractures and burns, may undergo intensive rehabilitation. The discomfort of therapy can reduce their compliance, limit their range of motion (ROM) and lead to chronic pain. Virtual Reality (VR) interventions have been found to reduce anticipated and procedural pain.

This feasibility study aimed to explore perceptions and impacts of a custom-made, fully immersive Head-Mounted Display VR (HMD-VR) experience within a United Kingdom (UK) National Health Service (NHS) outpatient rehabilitation service for children with ULIs.

**METHODS:**

Ten children aged 9–16 in one UK Children’s hospital trialled HMD-VR during one rehabilitation session. They, their parents (*n* = 10), and hospital physiotherapy staff (*n* = 2) were interviewed about their perceptions of pain, difficulty, enjoyability, therapeutic impacts, benefits, and limitations. Children rated the sessions on enjoyability, difficulty, and pain compared to usual rehabilitation exercises. Physiotherapists were asked to provide range of motion readings.

**RESULTS:**

Inductive thematic analysis of interview data generated three themes, ‘Escape through Engagement’; ‘Enhanced Movement’; and ‘Adaptability and Practicality’. Children rated the session as more enjoyable, less difficult and painful than their usual rehabilitation exercises. Findings suggested that HMD-VR was an engaging, enjoyable experience that distracted children from the pain and boredom of therapy. Also, it seemed to enhance the movement they achieved. Participants perceived it was useful for rehabilitation and adaptable to individual needs and other patient groups. Suggestions were made to increase adaptability and build in practical safeguards.

**CONCLUSION:**

Findings from this small-scale feasibility study suggested HMD-VR was perceived as usable, acceptable, and effective with potential for further development. Future work could include larger scale trials.

## 1 Introduction

Upper Limb Injuries (ULIs) in children, including fractures and burns, may require intensive and often painful rehabilitation. The annual UK population incidence of fractures is estimated at 3.6%. Based on circa 1.8 million fractures in England per annum in children and adults [1], the total annual cost to the NHS of fractures is between £3,168 million and £5,550 million [2]. Between 15 and 45% of children experience fractures [3] of which 28.6% involve upper limbs [4]. In a large-scale study of children’s burn injuries, most were due to scalds (58%) or contact burns (32%) [5]. For children age 5–16, most scalds were from spill injuries (76%) to the lower trunk, legs, and hands. Contact burns were mainly to the hands (67%) [5]. Thus, children with fractures or burn injuries may experience extended hospitalization and reduced physical activity, resulting in muscle weakness, and decreasing average lean mass of ULs compared to lower limbs [6]. Therefore, rehabilitation necessitates early mobilisation of ULs to ensure restoration of full functioning.

Rehabilitation therapy typically involves repetitive exercises to regain or maximise function of the UL and hands [5, 6]. The pain and discomfort of current therapeutic exercises can reduce compliance and, therefore, the range of motion patients achieve. This increases the risk of chronic pain and can reduce patients’ confidence in the care team [7]. Therefore, tackling rehabilitation pain is important in enhancing long-term outcomes for this group. Non-pharmacological pain relief methods aimed at reducing pain through distraction have been demonstrated as potentially effective in tackling the discomfort of painful procedures [8, 9].

VR is a computer-simulated three-dimensional environment which offers the user a multi-sensory experience in which their actions partially determine events [10, 11]. HMD-VR is a fully immersive form of VR, delivered by a head-mounted display unit which delivers sights and sounds, and in which users engage with the scenario using hand-held controls. HMD-VR is considered to reduce pain perception through its engagement of the user’s attention, emotions, concentration, and senses [12, 13].

In studies during physical therapy, VR interventions have demonstrated several benefits. They make additional demands upon the user’s attention demonstrated by increased activity in the premotor and motor cortices thereby distracting users from environmental cues to pain [14]. Adult burn patients reported significantly reduced pain and time spent thinking about pain during 3 minutes of physiotherapy compared with controls (no VR) [15]. In children, immersive VR can reduce pain and improve the experience of clinical procedures where pharmacologic analgesia alone is often inadequate. For example, immersive VR was used effectively in an 11-year-old child with 36% total body surface area burns of which 27% were full-thickness burns. VR was used in one of three monitored occupational therapy sessions during which pain intensity and unpleasantness respectively fell from severely to moderately painful and from moderately to mildly unpleasant; the child also reported being ‘completely inside’ the VR world and having fun [13].

As well as significantly reducing pain, VR has been found to reduce anxiety and improve care satisfaction for patients and caregivers during clinical procedures associated with anticipatory pain and anxiety, such as venepuncture for children and young people [12]. As well as reducing unpleasant effects of treatment, interactive VR can create positive effects for a user during interventions such as enjoyment and entertainment. This was demonstrated with child and adolescent cancer patients undergoing implantable venous access device needle insertion [16]. In that study, 82% reported finding the custom-made intervention easy to use; 94% found it understandable and wished to use it again during subsequent procedures.

Two systematic reviews [10, 17] of the application of VR interventions in clinical settings report wound dressings as the most commonly studied procedure with pain as a primary, and anxiety as a secondary measure. VR has been shown to reduce perceived pain for both adults and children during wound care [10, 18, 19]. However, although early evidence for VR came from burn research [15], there is growing evidence that VR can be an efficient and beneficial form of pain relief for children in rehabilitation [20–24]. For example, studies indicate that it shows greater potential than conventional therapy in motivating and improving UL functional outcomes in children with cerebral palsy without any negative effects [21, 22]. Jannink et al.’s randomised controlled pilot study [21] utilised EyeToy, a non-immersive screen VR game, to encourage elbow and shoulder movements. After three weeks of treatment, some children in the experimental group showed greater motion improvements than in the control group. Similarly, Sharan et al. [22] used the game platform Wii Sport and Wii Fit as a non-immersive VR-based therapy comparing the use of conventional therapy alone with combined VR therapy. After three weeks of playing the game on three alternate days each week, experimental group children showed a significantly greater improvement in balance scores, and significantly higher levels of participation, motivation, cooperation and satisfaction than the control group.

Kiper et al.’s randomised controlled trial with adult post-stroke patients [23] demonstrated the effectiveness of reinforced feedback in non-immersive screen VR treatment used for 2 hours daily, 5 days per week for 4 weeks, combined with conventional rehabilitation for upper limb rehabilitation, compared with conventional therapy alone. Finally, Pyk et al. [24] presented a non-immersive VR set of interactive scenarios for arm rehabilitation in children reporting that all patients accepted the system, and were trained in reaching and grasping tasks at a far higher rate than in conventional occupational therapy. Also, studies have also shown that VR training initially has a high intrinsic motivational power that increases participants’ level of engagement in and enjoyment of physical activities [21, 22, 25].

Much of the above evidence for VR in rehabilitation is based on non-immersive VR. It is important to determine the usability and acceptability of HMD-VR, monitoring factors such as comfort or nausea, particularly for users already experiencing discomfort or pain. Recent improvements in the technology of HMDs, making them lighter and untethered may allow children to participate in a more immersive and engaging VR experience [20]. However, with chronic pain patients, some studies [26] have reported that the head-mounted displays used in VR can cause simulator sickness for some users due to the design of the VR display layout. Despite occasional side-effects, immersive VR has been demonstrated as more effective than non-immersive VR for ULI motor recovery in adults after a stroke [27, 28]. This finding is supported by three systematic reviews, which show that VR-based activities for UL rehabilitation post-stroke in adults were more effective when compared with traditional care [29–31]. Taken together, these studies show a steady progression of evidence to support incorporating VR into UL rehabilitation interventions [32].

There is growing evidence for the effectiveness of VR in reducing procedural pain and its cost-effectiveness with very limited adverse effects [10]. VR has promising results for UL rehabilitation in adults [32]. However, few studies have tested immersive forms of VR, and none have tested HMD-VR technology during UL rehabilitation therapies for children in a clinical setting. Based on the perceptions of children, parents, and physiotherapists, this study set out to explore the user experience, therapeutic effect, and practical feasibility of two custom-made HMD-VR games interventions to support children’s UL physical therapy. It represents an early-stage, small-scale feasibility study of both the intervention and its implementation which provides qualitative evidence which could inform future larger-scale clinical trials. The choice of primarily qualitative methods is in keeping with a person-centred approach that has been recommended in developing healthcare interventions [33].

The study aimed to investigate:

• The perceptions of pediatric ULI patients, their parents, and practitioners of the impacts on pain, movement, enjoyability, and difficulty during one rehabilitation session of a custom-made HMD-VR intervention;
• And the perceived benefits, limitations, and potential of the HMD-VR intervention in children’s ULI rehabilitation therapy.

## 2 Methods

### 2.1 Review, funding, and approvals

Medical Research Council (MRC) funding (Grant number 152333/SCH2178/AA3179019) was awarded in August 2017 for intervention development, piloting and clinical trial work. Clinical data reported here were collected between 31st December, 2018, and 1st May, 2019. Ethical approvals were obtained from Sheffield Hallam University Research Ethics Committee, UK, and the Integrated Research Application System (IRAS, the approval system for health and social care research in the UK; project number 243763). Permissions were also gained from the host Children’s Hospital NHS Trust Research and Development department.

### 2.2 Participants

Participants were recruited from one Children’s Hospital Trust in England, UK. Children were eligible if they were aged 7–16 and had UL or hand injuries requiring rehabilitative care. Exclusion criteria included 1) injuries to the face or head that could hinder the correct positioning of the headset or pose an infection risk; 2) a learning impairment that could hinder the understanding of the task; 3) a history of severe motion sickness, and 4) mental health problems. Parents of eligible children, and the two physiotherapists involved in the usual care and HMD-VR trials were also invited to participate.

Physiotherapists involved in rehabilitation therapy screened and identified eligible participants and introduced the study. Parents and children were provided with age-appropriate information sheets. Both verbal and appropriate written consents were gained from children, as well as written consent from parents. Children’s ongoing consent was checked during the HMD-VR trial. A translator was provided for those with a first language other than English (*n* = 1).

Recruited participants included 10 children receiving ULI rehabilitation (aged 9–16, 4 boys and 6 girls), 10 parents and 2 physiotherapists who were present during the HMD-VR trials (see Table 1). ULIs in the sample were burns (*n* = 6) and fractures (*n* = 4). Affected UL areas included the elbows, hands, shoulders. Five children had injuries which affected several areas of the UL. One participating physiotherapist specialised in orthopaedic injuries and the second in burn-related injuries.

**Table 1:** Patient Demographic Information

Patient ID	Age in Years	Gender	Upper Limb Injury (ULI)	Clinician
pt1	10	Male	Burns sequelae (scar reconstruction arm, shoulder)	Physio2
pt2	10	Female	Nerve and muscle injury (head, arm, shoulder)	Physio2
pt3	10	Male	Burn Sequelae (Scar reconstruction arm, chest and shoulder)	Physio2
pt4	16	Female	Arm Motor Impairments (Wounds and Infection – Hidradermatitis suppraliva)	Physio2
pt5	10	Female	Arm Motor Impairments (Wounds and Infection – Hidradermatitis suppraliva)	Physio1
pt6	10	Female	Elbow fracture	Physio1
pt7	9	Male	Exostosis to lower arm	Physio1
pt8	9	Female	Elbow fracture and nerve palsy	Physio1
pt9	16	Male	Burns sequelae (scar reconstruction arm) trunk, head and face, arms and hands	Physio2
pt10	14	Female	Burns sequelae (scar reconstruction arm, wrist and hand)	Physio2

### 2.3 Equipment

**Fig. 1:**
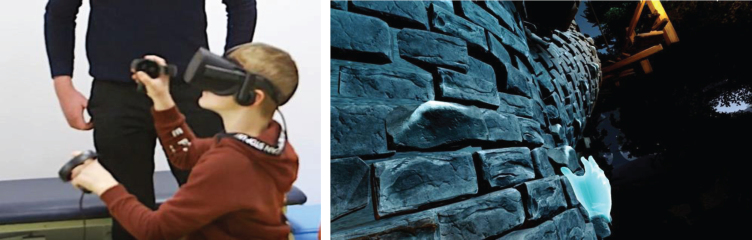
Images of Climbing Game.

**Fig. 2:**
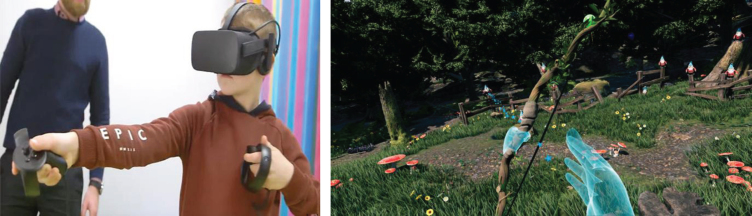
Images of Archery game.

Equipment included a VR headset with touch controllers, and software with two gaming environments using Unreal Engine 4.20, 3ds Max 2019, and Substance Designer 2018.3 software. Two custom-made HMD-VR games were developed by the games designer, IP, in consultation with physiotherapists and piloted with school children prior to the clinical trial. Games were designed to encourage the therapeutic movements required for ULI rehabilitation. In the Climbing game (Fig. 1) the child had to climb up to the top by performing an overhead arm raise exercise. Highlighted bricks and ropes were presented. Once the brick was grabbed the child lowered their arm to climb up. If the child failed to grab the brick, they fell off the climbing wall. To minimise discouragement after falls and preserve some previous progress, several checkpoints were developed for the child to land on at different levels. The Archery game (Fig. 2) involved using a bow and arrow to target balloons and gnomes. The child had to reach out with their non-injured arm to grab a bow floating in front of them, then lift up the injured arm and bend the elbow behind the back to grab an arrow from a quiver. Afterwards the child brought the arrow in line with the bow string to attach it, holding the bow outstretched and pulled back the injured arm holding the arrow.

### 2.4 Materials

Semi-structured interview schedules were developed (see Table 2). Children were asked to compare the pain, difficulty and enjoyment of the HMD-VR session with their usual rehabilitation session using a 0–10 rating scale (where 0 = much less painful/difficult/enjoyable than usual, 5 = the same as usual and 10 = much more painful/difficult/enjoyable than usual). Ratings helped provide a basis for more detailed qualitative questions and discussion with children and their parents about the pain, difficulty and enjoyability of the HMD-VR experience. Practitioners’ interview schedule included questions to explore perceived therapeutic effects, benefits, limitations and potential of the HMD-VR games. Questions were open and neutral to facilitate both positive and negative experiences. Digital audio recording equipment was used and external transcription services transcribed data.

**Table 2:** Interview Questions

Interview questions for children
•Enjoyment: Compared with how it is usually when you are doing your exercises, how much did you enjoy doing them today, with the game? (scale 0–10 where 0 is ‘much less enjoyable’ and 10 is ‘much more enjoyable’)
∘ Why did you give this answer? What things made the exercises more / less enjoyable? What was the best bit/worst bit of the game?
•Difficulty: Compared with how it is usually when you are doing your exercises, how difficult was it to make the movements? (scale 0–10 where 0 is ‘much less difficult’ and 10 is ‘much more difficult’)
∘ What made you give this answer? Were there times when you didn’t know what to do? Were any of the arm / hand / finger movements difficult to do? Can you show me what was easier / harder?
•Pain: Compared with how it is usually when you are doing your exercises, how painful or uncomfortable was it to do them today? (scale 0–10 where 0 is ‘much less painful’ and 10 is ‘much more painful’)
∘ How was it different this time? Which movements were more / less painful?
**Interview questions for parents**
•What were your impressions of how painful/enjoyable/difficult your child found the IVR game(s) and their exercises, compared to usual?
•In the future, is this something you could see being used at home?
**Interview questions for physiotherapists**
•Could you tell us a little about each of the children with whom you’ve used the IVR, and how it has worked in their sessions?
•We’d like to hear a bit more about the effects that you think the technology had on the children’s experience and their ability to do the exercises.
•How do you think seeing their child using the technology affected the parents who were present?
•What about IVR’s effect on you and your work with the children?
•How effective were the games in facilitating the exact movements the children needed to do during their exercises? Could you give some details about this?
•How do you think IVR affects physiotherapy in general?
•Apart from the children you used IVR with, how else could you imagine using the IVR in future?
•How might IVR be used as a home-based rehabilitation training?

### 2.5 Procedure

Participants’ usual experience of rehabilitation is a 20–30 minute appointment including a review of the previously prescribed home exercise regime, taking ROM readings via a goniometer, and an explanation and discussion of new exercises for the child to do at home. In this trial, in addition to usual care, participants were asked to try the two HMD-VR games. Afterwards, they are their parents were interviewed about their experience.

Sessions were attended by the child, their accompanying parent, the physiotherapist and IP, whose role was to assist with the technology and conduct the interviews. With support from IP, each child tried out the headset and had a tutorial for the first game (archery) before they and their parents gave consent for participating. All attending children and their parents wished to proceed.

Children played the archery game for 15 minutes, took a 5-minute break, and, following a short introduction (by IP) to the second game (climbing), played this for a further 15 minutes. During play, the physiotherapist observed the child’s movements, and, if required, adjusted VR height or distance settings to ensure each child was moving and being challenged appropriately. Parents did not play an active role, although occasionally they and physiotherapists offered encouragement to the child, for example, for successful shots or climbs. IP did not interact with the child but managed any technical issues and ensured the child’s safety within the environment during game play.

Following the sessions, IP conducted semi-structured interviews of 10–15 minutes, interviewing each child and their participating parent together in the rehabilitation setting. Children’s questions preceded those of their parents to avoid the possibility of parental responses influencing their children’s. The total session participation time for each child and their parents was 50–60 minutes. At the end of all child trials, PF conducted interviews of around 45 minutes with the two participating physiotherapists in a private meeting room. All interviews were audio recorded, transcribed, and analysed for themes [34].

### 2.6 Analysis

Interview data were analysed using inductive, semantic thematic analysis [34]. The 12 transcripts were read and re-read, coded alongside the text using a traditional pen-and-paper approach. Codes were then grouped based on apparent similarities. Spidergrams were used to reflect patterns in and relationships between grouped codes and emerging themes. Two authors conducted this coding process separately and met to agree on themes which were then considered and refined through team discussions. Descriptive statistical analyses appropriate to the small sample (range, median) were conducted on the numerical responses to the 0–10 scale questions.

## 3 Results

### 3.1 Children’s ratings of difficulty, enjoyment and pain

In comparison with their usual rehabilitation experiences, children reported finding rehabilitation exercises more enjoyable and movement easier and less painful using HMD-VR. Pain and difficulty were rated overall as lower than usual, both with median scores of 3 (pain range 0–6.5; difficulty range 0–7). Enjoyment was rated as much higher than usual, with a median rating of 10 (range 8–10).

### 3.2 Goniometer ROM readings

Physiotherapists routinely recorded patients’ ROM readings during rehabilitation and were asked to provide pre- and post-VR goniometer data for the children in the study after its completion. Unfortunately, only partial data could be retrieved. There were some discrepancies between participants in the timing of readings, with some taken on the day of VR and others in prior or subsequent rehabilitation sessions in which limitations rendered statistical analysis unreliable. However, readings did indicate some positive changes in ROM for 5 children, as shown in Table 3.

**Table 3:** Descriptive ROM Data (Physio2 Readings Only)

Patient ID and injury	Upper limb joint affected	Joint movement	ROM (degrees)	
			Pre-VR	Post-VR
Pt1: Burns sequelae	Shoulder	Flexion	180	180
		Extension	65	65
		Abduction	180	180
		Adduction	30	30
Pt2: Nerve and muscle injury	Shoulder	Flexion	130	165^*^
		Extension	50	60^*^
		Abduction	100	135^*^
		Adduction	30	65^*^
		Medial rotation	20	75^*^
		Lateral rotation	80	90^*^
	Elbow	Flexion	130	145^*^
		Extension	0	0
Pt3: Burn Sequelae	Shoulder	Flexion	160	165^*^
		Extension	80	70
		Abduction	120	175^*^
		Medial rotation	90	65
		Lateral rotation	70	90^*^
Pt4: Arm Motor Impairments	Shoulder	Flexion	130	170^*^
		Abduction	100	170^*^
Pt9: Burns sequelae	Elbow	Flexion	140	144^*^
		Extension	–30	–30
	Wrist	Flexion	10	13^*^
		Extension	80	75
		Supination	80	82^*^
		Pronation	85	90
Pt10: Burns sequelae	Elbow	Extension	–20	0^*^
	Wrist	Flexion	50	45
		Extension	45	70^*^
		Supination	75	90^*^
		Pronation	90	90
		Radial Deviation	25	25

*Notes.* ROM = Range of Motion in a joint, based on goniometer readings. ^*^indicates an increase in ROM between pre- and post-VR readings.

### 3.3 Qualitative themes

Interview data were anonymised, and participants referred to below as, e.g., pt1, pt2 (child patients); pt1P, pt2P (parents); and Physio1, Physio2 (physiotherapists). Three themes were generated from the analysis of interview data: ‘Escape through Engagement’; ‘Enhanced Movement’; and ‘Adaptability and Practicality’.

#### 3.3.1 Escape through Engagement

Using HMD-VR game scenarios during rehabilitation offered children an escape from the usual negative experiences associated with rehabilitation therapy such as boredom or pain and produced positive emotions along with increased engagement with exercises. Occasional frustrations did not impair engagement, as it might have under usual therapeutic conditions. Engagement was supported by games which offered an appropriate level of difficulty and challenge.

Although participants acknowledged that rehabilitation was necessary for recovery, it was seen as boring. Children were unmotivated to engage fully (e.g., ‘*I don*’*t like repeat it (exercises), but like it does help me*' (pt5)). In addition, some children perceived rehabilitation as painful, which hindered their engagement, for example: ‘*When I do my bending exercises, when I bend it more, it sometimes hurts*’ (pt7).

In contrast and in keeping with children’s ratings, during and immediately after the HMD-VR trial, most participants reported children experiencing less pain and being more motivated than after usual treatment:

’*It was much, a bit more, easier than I normally do’* (pt7). 

‘*I didn*’*t notice any pain’* (pt7P). 

‘*He didn*’*t want to do any exercises after the operation and now with the game, he just wants to do it’* (pt1P). 

Not only did the HMD-VR reduce the negatives of rehabilitation, but it also evoked positive feelings and emotions towards the games and rehabilitation exercises in all of these children:

“*It was really fun... and then the best bit was the climbing one where I got like near to the top*” (pt5). 

“*I just found them both really good. I really enjoyed them... I liked shooting the balloons... because it*’*s like*... *it was quite magical, and how they exploded into lots of colour*” (pt6). 

Parents and physiotherapists made similar observations about boredom, pain and enjoyment:

‘*It was obviously much less boring than doing the same thing over and over again, even though she probably was doing the same range of movements*’ (pt8P). 

HMD-VR seemed to help children to escape into a ‘virtual’ world of exciting distractions that helped them to forget the negative experiences of therapy:

‘*I was distracted, because I wasn’t paying attention to it [injury]*’ (pt2). 

‘*It’s more like magical, it’s like it just makes you feel a bit excited*’ (pt6). 

The children’s enjoyment had positive effects on the physiotherapists and parents present:

‘*He* (pt7) *enjoyed that more than following a sheet of instructions. I felt pleased that children enjoyed and were motivated, I saw their excitement and them engaging in a different sort of activity*’ (Physio1). 

*’One moment, the mum got a bit tearful but in a nice way. Like happy, I can’t believe she’s doing this way. I was surprised at that, actually, at how much movement they did get when they were immersed in something else, and that, I think, it’s the big difference. So, I was proud before, I’m very proud now. I think it is brilliant, thank you very much’* (Physio2). 

The HMD-VR games also stirred some less positive emotions and sensations that could otherwise impede children’s engagement with exercises, such as frustration while developing expertise with game features. Compared with usual therapy, however, with HMD-VR, children were motivated to continue despite these experiences: ‘*It was kind of frustrating because I kept on dropping my bow, but I like popped some balloons and some gnomes so, it was really fun*’ (pt5). Physiotherapists were surprised that patients who usually withdrew from therapy when frustrated continued to engage positively when frustrated by the games: ‘*I was surprised he carried on, but he did carry on, and did then really enjoy it*' (Physio2).

Only one patient reported that the exercises were more difficult than usual exercises, citing extra stretching with HMD-VR: ‘*It was harder than the normal exercises, moving around more, do some stretch more*’ (pt8). The majority said HMD-VR made the exercises easier, often because they were focusing on the game, rather than what they were doing with their arms: ‘*It was easier because I did not pay attention to it*’ (pt2). The level of difficulty of the two HMD-VR games was considered appropriate by all children in this sample, irrespective of age, gender or ULI type. Being challenged at the right level appeared to increase children’s motivation to exercise:

‘*It was quite fun, but they were difficult. And it made me enjoy them a bit more that they were difficult*’ (pt6). 

#### 3.3.2 Enhanced movement

Escaping from boredom and pain, and engagement with enjoyable, challenging HMD-VR games seemed to enhance children’s movement during therapy. Participants spoke of natural movements, greater tolerance, effort, duration and range of motion, and the benefits of HMD-VR for stamina and confidence.

Using HMD-VR games during rehabilitation promoted more natural and fluent movements than usual therapy by distracting patients so they moved without thinking:

’*It was just so fluent, she didn’t have to think about what she was doing*’ (pt2P). 

*’The amount of movement you got was a lot more than [the physiotherapist] told me you can get in your arm. So, I think it probably was a lot easier, doing it more naturally without thinking’* (pt4P). 

‘*The fact that she could move the whole of her body I think is really important*’ (pt8P). 

Perceived therapeutic effects were positive: children’s engagement, effort and tolerance of exercises during rehabilitation were considered greater using HMD-VR than under usual conditions:

‘*I think it*’*s much more engaging and the effort they put into the movement is much greater than you expect from normal exercise. And certainly, enjoyment as well is, you can see that they*’*re benefiting from being fun’* (pt6P). 

’*we were very caught up in the activity, and they were distracted from what we were doing. It extended the length of time they did an exercise’* (Physio1). 

‘*I’ve never seen him do his movements quite so happily in many years*’ (pt9P). 

In keeping with their goniometer data, physiotherapists reported that the children in the sample increased their range of motion during the one session of HMD-VR, including those whose performance had previously plateaued:

*’We take measurements of their joint ranges and actually he did gain some movement during the session, but I wasn’t expecting him to, to be honest ‘cause he’s been fairly static with what he’s got, movement wise’* (Physio2). 

*’The girl with [specifies injury] got a huge amount of more range of movement has significantly improved just with that one session’* (Physio2). 

Given the increased effort, engagement and duration, physiotherapists noted some tiredness after the HMD-VR session:

‘*Some of them have said their arms have been quite tired or a bit achy because they’ve done more than they would normally, and their arms been up for longer, but no actual pain and discomfort at all*’ (Physio2). 

However, physiotherapists also observed that the post-trial discomfort was not excessive or therapeutically problematic. Indeed some discomfort was expected and desirable as a means of building stamina:

‘*From a tolerance and a stamina point of view (using HMD-VR) they were having to hold their arm up. (That will) build up power, to make sure they don*’*t get a stiff shoulder*’ (Physio1). 

Moving freely without experiencing pain within the HMD-VR game was considered to increase children’s confidence and positive engagement with URT exercises:

‘*(Her)* (pt4) *range of movement has significantly improved just with that one session, because it got over the confidence to realise that her arm could do that*’ (Physio2). 

‘*It*’*s a very good thing from the psychological point of view in the children, them feeling good about themselves and achieving something that they maybe thought they wouldn’t be able to do’* (Physio2). 

#### 3.2.3 Adaptability and practicality

This theme describes participant perceptions that HMD-VR could make a positive impact on rehabilitation with this patient group and be adaptable to other contexts. It was considered to have potential in increasing engagement with and reducing the isolation of patients undergoing long-term rehabilitation at home. In the context of problematic gaming behaviour, physiotherapists saw the scope for further adaptability to the intervention and identified practical safeguarding considerations to prevent over-exercise, protect expensive equipment.

Participants perceived the HMD-VR intervention and its future potential very positively:

’*The game is very suitable for the exercises, for the kind of exercises he needs for his arm*’ (pt1P). 

*’I would feel very positive yeah, be happy, yeah, (for it) to be part of our therapy’* (Physio1). 

HMD-VR provided clinicians with an additional rehabilitation therapy tool and alternative ways to assess and positively interact with patients.

*’There probably was a bit less communication than there would have been normally during a session, but there was a lot of positive communication as well’* (Physio2). 

*’It’s a lot less hands on for us. It gives you the opportunity to sit back and have a good look at what they’re doing a bit more. I was watching them, I was picking up, sort of, how much they were moving and assessing them all the time when they weren’t aware of it’* (Physio2). 

Participants also envisaged HMD-VR being used in patient cohorts beyond pediatric rehabilition. Suggestions included adult patients and people with acute or chronic conditions, lower limb or spinal injuries, and neurological conditions:

*’And it would be quite good from a lower limb stance, you know, standing tolerance and weight shifting from a spinal injury or those sorts of conditions, it would be quite useful too’* (Physio1). 

*’I could see it used pretty much across all the areas that we see patients across the hospital cause there’s something in it for everybody’* (Physio2). 

Two (pt5P and Physio1) expressed concern that children’s enthusiasm for playing HMD-VR games may lead to over-exercise, causing pain or inflammation to injury sites; however, none was reported as a result of the present trial. Indeed, some felt HMD-VR could reduce the amount of exercise sessions needed:

*’So, they do six reps six times a day (at home) for simple exercises. I wouldn*’*t expect VR to be used six times a day but if they were using it once a day, maybe, varies depends on injury. (In clinic) minimum time, it*’*s half an hour’* (Physio1). 

’*We’d assess each child individually and what their capacity were, but some children 5*–*10 minutes might be enough on a daily basis for them’* (Physio2). 

Data suggested HMD-VR could motivate children to engage with long-term rehabilitation therapy and promote positive patient/carer relationships in both clinical (e.g., hospital) and non-clinical settings (e.g., at home or school):

*’They (parents) were all saying that it was, there wasn’t any arguments with it; they (children) were all engaged and wanting to do it. It made it a lot easier - the parents that have to do the stretches on the kids were really positive about the fact that they were achieving that amount of movement without them having to do anything ‘cause it’s a bit of a battle at times*... *the impact of that on the family must be very draining, and it must affect your relationship as a parent and child when you are constantly battling to get them to do things’* (Physio2). 

Discussing future developments, clinicians spoke about the potential for interactive HMD-VR to increase social engagement, reduce the isolation often experienced by children who are undergoing lengthy rehabilitation, and to promote psychosocial well-being. This might involve playing prescribed HMD-VR games with people with the same condition, or friends and family:

*’You could maybe have it (HMD-VR) as more of an interactive thing where they’re playing with other people as well, I think from a social and psychological point of view, that’d be really useful, being able to do their therapy with their friends, like, have little competitions, and keep each other going and chat through it.’* (Physio2). 

These two HMD-VR game scenarios and hardware were positively received because of their versatility, an essential feature when individualising therapeutic exercises for patients. Physiotherapists appreciated being able to remotely alter the system for each child and stressed the importance of its adaptability to their needs: ‘*So, you would always adapt anyway and tailor-make your therapy to that child. So, yes, it (HMD-VR) needs to be adaptable’* (Physio1).

With adaptability in mind, physiotherapists wanted a wider choice of games/activities that: have varied levels of difficulty *(’choose the difficulty level you go for, might be useful’* (Physio 2)), promote a range of movements (’*We were going from seven to 16, so a seven-year-old is gonna need a very different range of movements to a 16-year-old’* (Physio 2)); and promote children’s normal activities of daily living (’*Link to more practical activities’* (pt5P)). Altering the system to use additional button presses was suggested for children with thumb fracture injuries.

To facilitate individualised prescribing, prevent over-exercise and promote therapeutically desired movements, physiotherapists suggested that future HMD-VR interventions could include some additional practical safeguards. These included offering ‘*a recommendation of what we thought was an appropriate amount of time*’ (Physio2), and time-outs for therapeutic reasons: ‘*so they can only use for a certain amount of days’* (Physio 2) or ‘*to limit their screen time and their technology time’* (Physio1), as well as controlled access, loan deposits, lockable trolleys and bar codes to reduce the likelihood of thefts. The two physiotherapists involved in this study stressed the importance of staff training to motivate and ensure best practice in the use of the technology with clients.

## 4 Discussion

This small-scale study explored child user, parent and physiotherapist perceptions of enjoyability, difficulty, pain and movement during a rehabilitation session using HMD-VR game scenarios, and the perceived potential, benefits and limitations of HMD-VR in paediatric rehabilitation therapy following ULIs. Findings from qualitative interview data suggest HMD-VR increased children’s motivation and confidence to engage with therapeutic exercises and range of motion achieved compared to usual therapy. HMD-VR increased children’s engagement by eliciting positive emotions, concentration, and sensory involvement. This distracted children, enabling them to escape their usual therapy experiences of pain, discomfort, and boredom. Positive emotions were stimulated by the challenges, and a sense of achievement and control encountered within both HMD-VR games, which all participants perceived as missing from children’s usual therapy and daily activities. This finding supports the hypothesis that distraction is the mechanism in VR that mediates pain [35], and its applicability in this cohort.

All participating children, regardless of age, sex, culture or ULI, found both HMD-VR games physically and cognitively challenging yet achievable with support from adults: i.e., the researcher provided instructions on how to use the HMD-VR technology, the physiotherapist made corrective postural adjustments, and parents offered encouragement. Such adult support is consistent with Vygotsky’s [36] sociocultural theory that suggests that children’s performance is greatest in ‘the zone of proximal development’ where the child’s current level of performance (e.g., range of motion achieved), and potential performance is promoted through stretching the child both mentally and physically with adult guidance. Present findings suggest tensions often exist in the relationship between children (patients) and adults (clinicians and parents) due to children’s resistance to usual rehabilitation exercises that all considered boring and repetitive. Such conflict is integral to care giver/care recipient relationships. For positive relationships to flourish, care recipients’ independence and dignity must be maintained [37].

Children trialling HMD-VR games in this study were empowered and motivated to engage independently in rehabilitation exercises which were perceived to have improved their clinical outcomes. The low cost and apparent therapeutic benefits of the HMD-VR technology trialled here supports the suggestion made by World Health Organisation that affordable technology could address barriers to rehabilitation globally [38]. Physiotherapists saw HMD-VR as a tool that increased opportunities to correct patients’ movements, and perceived that the positive ambiance it created enhanced communication with patients and their parents. HMD-VR also injected a sense of fun and laughter into outpatient rehabilitation exercise sessions that improved the experience for children and, unexpectedly, for parents and physiotherapists. This positive experience was considered replicable at home and in the community, again under adult supervision, and with suitable adjustments custom-made to the biopsychosocial and cultural needs of individuals. This could include prescribing games that stimulate required movements and designing game scenarios to avoid undesirable images such as guns or, for burn patients, flames. Present findings highlight: the importance of physiotherapist and parental support in children’s rehabilitation journeys; HMD-VR’s potential to reduce inherent tensions in the care giver/care-recipient relationship; and the sociocultural influences in children’s ongoing recovery from ULIs. Thus, future research to develop, trial, and integrate HMD-VR within existing children’s ULI rehabilitation services may be enhanced by drawing on sociocultural theory [36].

The current findings were based on a single session, and it could be argued that the novelty factor was responsible for some outcomes. However, some previous studies have shown the effectiveness of VR to improve children’s motivation and clinical outcomes where the treatment duration varied from a one-day session of 12 minutes to 20 weeks [39]. The two HMD-VR games trialled here were positively received. However, additional modifications were suggested by participants, aimed at empowering clinicians to prescribe appropriately and safely, and children to engage fully over time. Ideas included: more game scenarios and levels of difficulty; clinician control over patient access and game usage; controllers with relevant button presses for use in thumb fracture injuries; staff training on the use of HMD-VR technology; and durability and security of hardware. The readily available and relatively inexpensive hardware used in the study was both usable and acceptable to all patients, and none reported negative effects such as nausea [26]. Indeed, VR technology could be a clinically beneficial and cost-effective treatment option if patients have their own head-mounted display (HMD) at home [40]. One that could facilitate frequent home-based exercises may aid in pain management and enhance duration/range of motion.

Both HMD-VR game scenarios and hardware were also perceived to have high usability and acceptability, not only within the present cohort, but also for other patient groups (e.g., neurology; spinal injuries; LLIs) and contexts (e.g., at home and in the community). The former is supported by an increasing body of literature supporting the use of HMD-VR for adults and children across a range of conditions and therapeutic interventions [10, 17]. The latter is supported where entertaining computer game-based interventions were found to increase children’s motivation for and compliance with rehabilitation exercise at home [41]. Of particular note were participants’ suggestions that multiple-user games could facilitate wider social engagement, particularly for patients with chronic conditions or long-term treatments which, based on sociocultural theory [36], have the potential to improve children’s rehabilitation performance.

These children reported experiencing less pain or discomfort using HMD-VR than during usual therapy which is consistent with previous immersive VR research, for example, during occupational therapy with paediatric burn patients [13, 42], and using virtual reality hypnosis with paediatric fracture patients [43]. The distress associated with pain can impact negatively on patients’ physical and psychological outcomes including limiting their ROM during ULI therapy [44], increasing their risk of Post-Traumatic Stress Disorder after a burn injury [45] or reducing confidence in the care team [7]. These physiotherapists instead reported increased ROM in all children using HMD-VR, a finding consistent with other work [46].

The shortcomings of the present study include the small number of participants which is typical of single-site trials. Two physiotherapists were enthusiastic; however, other clinicians such as medical and surgical staff may have provided alternative perspectives. The children in this study were exposed to a single session of VR so it is impossible to say whether the positive impressions of our participants would persist over time. Previous findings are mixed, with some suggesting that positive effects are maintained [39] and others, observing that repeated use of VR may result in fading interest and reduced effectiveness [47]. Limitations include the lack of baseline or control data and missing or inconsistently collected ROM readings. Collecting qualitative interview data provided depth and scope for unanticipated findings; however, they are not intended to be generalizable or representative. Children and parents were interviewed together for ethical and pragmatic reasons. There is a chance that responses may have been different, were children interviewed separately from parents; however, children’s questions preceded parents’ to limit the possibility of bias.

Present findings corroborate pain theories such as Neuromatrix Theory and Gate Control Theory [48]. Both emphasize a biopsychosocial and multidisciplinary pain approach [49], and the importance of psychological aspects such as attention, anxiety and perception [11, 50]. Future research should include measures of biopsychosocial determinants, psychological variables (e.g., motivation) and clearer clinical outcomes (e.g., ROM) using theory-based measures (e.g., validated questionnaires) or measures recognised and used in current clinical practice (e.g., NICE guidelines) [35]. Research should also involve larger samples, consider the perspectives of the broader clinical team and address the effect of repeated use of VR and the durability of the analgesic effect and user engagement with it. The one longitudinal study which has investigated the relationship between VR treatment and pain (albeit chronic pain, which is arguably a more complex issue) found that, though VR treatment was more effective than non-VR treatment, its effect had not persisted by the end of the five-week intervention period [47].

## 5 Conclusion

Overall, findings in this small-scale feasibility study suggest that, based on a single physiotherapy session, the HMD-VR intervention was positively perceived in terms of engagement, enjoyability and difficulty with positive impacts on pain and movement, and, with additional adaptation, considerable future potential in rehabilitation following ULI. This study adds to a small but growing body of research suggesting that HMD-VR is beneficial in paediatric rehabilitation; however, additional research and larger-scale and longitudinal trials are required to fully test its effectiveness in the clinical population.
